# Gold Nanoprobes for Detection of a Crucial EGFR Deletion for Early Diagnosis of Non-Small-Cell Lung Cancer

**DOI:** 10.3390/bios14040162

**Published:** 2024-03-29

**Authors:** Maria Enea, Anupong Nuekaew, Ricardo Franco, Eulália Pereira

**Affiliations:** 1 LAQV/REQUIMTE, Departamento de Química e Bioquímica, Faculdade de Ciências, Universidade do Porto, Rua Campo Alegre, 687, 4169-007 Porto, Portugal; anue279@aucklanduni.ac.nz (A.N.); eulalia.pereira@fc.up.pt (E.P.); 2 Associate Laboratory i4HB, Institute for Health and Bioeconomy, Faculdade de Ciências e Tecnologia, Universidade NOVA de Lisboa, 2819-516 Caparica, Portugal; 3 UCIBIO, Applied Molecular Biosciences Unit, Departamento de Química, Faculdade de Ciências e Tecnologia, Universidade NOVA de Lisboa, 2819-516 Caparica, Portugal

**Keywords:** gold nanoprobe, DNA detection, EGRF mutation, non-cross linking, UV-Vis

## Abstract

Gold nanoparticles (AuNPs) exhibit improved optical and spectral properties compared to bulk materials, making them suitable for the detection of DNA, RNA, antigens, and antibodies. Here, we describe a simple, selective, and rapid non-cross linking detection assay, using approx. 35 nm spherical Au nanoprobes, for a common mutation occurring in exon 19 of the epidermal growth factor receptor (EGFR), associated with non-small-cell lung cancer cells. AuNPs were synthesized based on the seed-mediated growth method and functionalized with a specific 16 bp thiolated oligonucleotide using a pH-assisted method. Both AuNPs and Au nanoprobes proved to be highly stable and monodisperse through ultraviolet-visible spectrophotometry, dynamic light scattering (DLS), and electrophoretic light scattering (ELS). Our results indicate a detection limit of 1.5 µg mL^−1^ using a 0.15 nmol dm^−3^ Au nanoprobe concentration. In conclusion, this work presents an effective possibility for a straightforward, fast, and inexpensive alternative for the detection of DNA sequences related to lung cancer, leading to a potential platform for early diagnosis of lung cancer patients.

## 1. Introduction

Gold nanoparticles (AuNPs), known for their biocompatibility and stability, find extensive applications in drug delivery and biosensing [1,2,3,4]. Their capacity to directly conjugate with various biomolecules, including proteins, drugs, antibodies, and nucleic acids, enhances the potential for diverse biomedical applications. The high surface-to-volume ratio of AuNPs facilitates effective conjugation, and their localized surface plasmon resonance (LSPR) sensitivity to size and shape enhances the performance of colorimetric biosensors. Recently, the exponential increase in the use of AuNPs in sensor development has led to improved sensitivity and selectivity, as well as simplification, of analysis procedures, revolutionizing biodetection methods [5,6,7,8].

Lung cancer is a prevalent form of primary malignant tumors leading to high mortality rates globally [9,10]. In recent decades, the number of deaths attributed to lung cancer worldwide has exceeded one million annually [10]. In many countries, the number of deaths from lung cancer exceeds those caused by other common types of cancer, mainly due to the absence of detectable symptoms. The clinical diagnosis of lung cancer heavily relies on expensive and invasive imaging techniques such as computed tomography (CT), magnetic resonance imaging (MRI), X-ray, and positron emission tomography (PET) [9]. These methods are costly and present challenges in terms of speed, non-invasiveness, and early detection. Furthermore, they can have adverse effects on human health and are inaccessible for low-cost screening, with direct impact on the survival outcome [9,11]. Consequently, there is an urgent need to complement the existing methods with simpler, less expensive, and less invasive methods.

Non-small-cell lung cancer (NSCLC) is the most common type of lung cancer [12]. Researchers have found a promising approach to treat NSCLC by targeting epidermal growth factor receptor (EGFR) in patients. Tyrosine kinase inhibitors (TKIs), such as gefitinib or erlotinib, have been used and have shown effectiveness in curing patients associated with this type of cancer [13,14]. Specifically, NSCLC patients with certain mutations in the EGFR protein have shown very good responses to TKIs [15]. The most common mutations in the EGFR gene occur in specific parts of the gene, called exons 18–21. These mutations, frequently found in NSCLC patients, include deletions in exon 19 and a specific change known as the L858R mutation in exon 21. These mutations are strongly associated with a better response to TKIs in lung cancer patients [16]. Recent studies have shown that lung cancer patients with the exon 19 deletion mutation tend to have longer survival than those with the L858R mutation when treated with drugs like gefitinib or erlotinib [17]. Knowing the mutation state of EGFR is, thus, essential to predicting how well a patient will respond to TKIs.

The current method of directly sequencing exon 18–21 of the EGFR gene from cancer samples is time-consuming and expensive due to the need for multiple steps and DNA amplification. Thus, it is critical to develop effective and fast methods to detect these mutations. A promising strategy is to use detection methods based on the optical and aggregation properties of AuNPs that are highly affected by the size and shape of AuNPs [5,18,19,20]. Among many morphologies and sizes, spherical 35 nm AuNPs are expected to present increased detection sensitivity, as they present a high extinction coefficient, and therefore a more intense color than the most common 15 nm AuNPs. In addition, the lower curvature of these AuNPs is expected to increase the number and stability of interactions with the target [5]. The present work is focused on developing 35 nm Au nanoprobes for the optical detection of an EGFR mutation associated with lung cancer. For this purpose, AuNPs were functionalized with a thiol-modified oligonucleotide, and the Au nanoprobes were tested using a non-cross-linking approach for discrimination among different 84–100 bp long synthetic DNA targets.

## 2. Materials and Methods

### 2.1. Au Nanoprobe Synthesis

#### 2.1.1. Au Nanoparticles Synthesis and Characterization

All reagents used in the study were of high purity or analytical grade and purchased from Sigma-Aldrich (St. Louis, MO, USA) or Merck (Darmstadt, Germany). Synthesis of the spherical-shaped AuNPs (35 nm) was performed by a seed-mediated method described by Bastus et al. [21]. First, a seed solution was prepared by addition, under heating and continuous stirring, of 1 mL of 25 mmol dm^−3^ HAuCl_4_ to 150 mL of 2.2 mmol dm^−3^ sodium citrate solution, and the mixture was refluxed for 10 min. Then, the mixture was cooled to 90 °C, and three growth steps were performed with the addition of 1 mL of 25 mmol dm^−3^ HAuCl_4_ and refluxing for 30 min with continuous stirring. The resulting AuNPs-containing solution was characterized by UV-Vis spectroscopy. The concentrations and diameters of the obtained AuNPs were calculated using the method of Haiss et al. [19] Concentrations of the stock suspension of AuNPs were 0.22 nmol dm^−3^. Hydrodynamic diameter and size distribution were evaluated by dynamic light scattering. All stocks of AuNPs were routinely checked using UV-Vis spectroscopy and DLS-ELS in order to evaluate their colloidal stability. If any changes in their stability were noticed, either visually or through modifications in their UV-Vis spectra or Z average and/or Zeta potential values, those samples were discarded, and fresh synthesis was performed.

#### 2.1.2. Functionalization

All ssDNA used in the current study (unmodified target DNA and thiol-modified ssDNA oligonucleotide) was purchased from STAB Vida, Lda. (Portugal). The thiol modification of the oligonucleotide was located at the 5′ end (Table 1). The functionalization of the AuNPs was performed based on a pH-assisted method [22,23] and 10 different oligonucleotide:AuNPs ratios were used: 250, 500, 800, 1000, 1250, 1500, 1750, 2000, 2250, and 2500 [22,23]. Briefly, 500 µL of 35 nm AuNPs were concentrated by centrifugation at 800× *g* for 12 min, and the resulting AuNPs were mixed with the oligonucleotide at the desired ratio. Following a 1 h incubation period, 8 µL of a pH 3 citrate/citric acid buffer solution with a concentration of 500 mmol dm^−3^ was slowly introduced and allowed to incubate for an additional hour. Subsequently, the mixture underwent centrifugation at 800× *g* for 10 min, followed by resuspension in a 10 mmol dm^−3^ phosphate buffer solution with a pH of 8. All solutions were stored in darkness at a temperature of 4 °C. UV-Vis spectroscopy, along with characterization using DLS and gel agaroses, was regularly conducted during the optimization process.

### 2.2. Au Nanoprobe Characterization

#### 2.2.1. Gel Electrophoresis

Agarose was from Invitrogen (Thermo Fisher Scientific, Waltham, MA, USA), and electrophoresis equipment was from Bio Rad (Hercules, CA, USA). A 0.3% *w*/*v* agarose gel was created by dissolving agarose in a 1:8 ratio of TAE buffer with a pH of 8.0. Once the gel had solidified, 10 µL samples were applied to each lane, and the gel was electrophoresed at 120 V for 20 min using the same running buffer. Following the electrophoresis run, images of the gel were captured using an Apple iPhone 11 camera to document the positions of the bright red bands derived from AuNPs.

#### 2.2.2. UV-Vis Analysis

Attenuance spectra for all samples were obtained using a Genesys 10S UV-Vis spectrophotometer (Thermo Scientific, Waltham, MA, USA). All spectra were obtained at an ambient temperature over a wavelength range of 400 to 900 nm, utilizing quartz cells with a path length of 1 cm (Hellma, Munich, Germany). Analysis of the AuNPs stock involved preliminary dilution 4 times with Milli-Q water. Unless specified otherwise, all nanoprobe samples were diluted in a 10 mmol dm^−3^ phosphate buffer with a pH of 8 before measurement, resulting in a final concentration of 0.15 nmol dm^−3^.

#### 2.2.3. Dynamic Light Scattering and Electrophoretic Light Scattering

A DLS-ELS: Zetasizer Nano ZS (Malvern Panalytical, Malvern, UK) instrument with light detection at 173° (DLS) and 17° (ELS) was used for the measurements of zeta potential and hydrodynamic diameter at 25 °C. Five measurements were taken for each sample, and solution dilutions and buffer conditions were as in the UV-Vis analysis described in Section 2.2.2.

### 2.3. Non-Cross-Linking Detection Assay

The Au nanoprobes, with a final concentration of 0.15 nmol dm^−3^, were mixed with synthetic DNA targets in 10 mmol dm^−3^ phosphate buffer (pH 8). Three types of DNA targets were employed: a fully complementary sequence to the Au nanoprobe (normal DNA), a mutated and deleted DNA sequence that lacked complementarity to the nanoprobe, and a completely random non-complementary sequence to the nanoprobe. The concentrations of the target DNA used in the experiment ranged from 1.5 to 36 µg mL^−1^. The assay mixtures were incubated at 39 °C and then allowed to cool to room temperature for 10 min to optimize hybridization. Subsequently, MgCl_2_ was introduced and left for 10 min at room temperature to initiate aggregation of the Au nanoprobes in the presence of various DNA targets. Visual inspection and UV-Vis spectroscopy were employed to analyze all samples. A blank sample containing only the Au nanoprobe and MgCl_2_ salt at the corresponding concentrations (without any DNA target) was used, along with another control labeled “Au nanoprobe” containing the Au nanoprobe alone (without salt or DNA target).

The Abs_λnon-aggregated_/Abs_λaggregated_ ratio was calculated for all spectra, as follows: Initially, the extinction values corresponding to the minimum and maximum were calculated for the spectrum resulting from the subtraction of the spectrum of the aggregated sample (blank control) from the non-aggregated sample (Au nanoprobe). The minimum value indicated the wavelength associated with the non-aggregated state, while the maximum value represented the wavelength linked to the aggregated state. These wavelengths were subsequently utilized to calculate the ratio between the Abs_λnon-aggregated_ (extinction peak of the non-aggregated nanoparticles (minimum)) and Abs_λaggregated_ (characteristic extinction peak of the aggregated nanoparticles (maximum)), which was associated with the colorimetric response for each sample.

### 2.4. Statistical Analysis

Statistical analyses were conducted utilizing GraphPad software Version 9.5.0 (GraphPad Software, San Diego, CA, USA). A minimum of three independent experiments were taken for each sample, and the results are expressed as mean ± standard deviation (SD). For the assessment of the normality of the data distribution, the Kolmogorov–Smirnov, D’Agostino and Pearson, and Shapiro–Wilk tests were used, and the comparison between normal DNA samples and the mutated/deleted DNA samples was performed using the unpaired Student’s *t*-test. For all comparisons, the significance was considered starting with a *p* value < 0.05.

## 3. Results and Discussion

**Figure 1:**
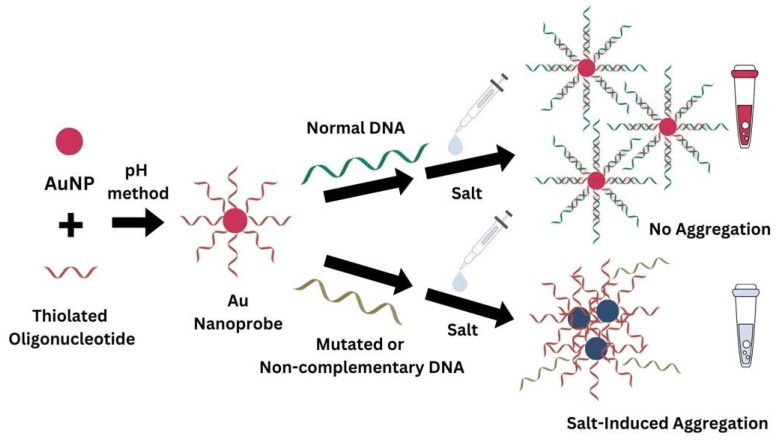
The detection assay was based on the aggregation state of Au nanoprobes in the presence of target DNA, which was complementary or non-complementary (deleted), or in the absence of target DNA (blank) after addition of salt.

The discrimination assay between complementary (normal) and mutated/non-complementary single-stranded DNA (sDNA) was based on the scheme from Figure 1. A 16-mer ssDNA, used for the functionalization of the AuNPs, was fully complementary to the DNA sequence corresponding to the normal target and non-complementary to the DNA sequence featuring an exon 19 deletion. After incubation of the Au nanoprobes with DNA targets and controls, the resistance to aggregation induced by salt addition was evaluated using UV-Vis spectroscopy. The anticipated outcomes were as follows (refer to Figure 1): (i) When hybridized with entirely complementary DNA (normal), Au nanoprobes exhibited resistance to salt-induced aggregation, and no significant changes could be seen in the corresponding plasmon band. (ii) Conversely, for the other samples, including the blank (absence of DNA target), the deleted DNA target, and the negative control (non-complementary DNA), Au nanoprobes presented aggregation after salt addition. This caused a color change from red to blue, associated with the emergence of a new localized surface plasmon resonance (LSPR) band at higher wavelengths (approximately 700 nm). (iii) The ratio between the absorbance at the wavelength corresponding to the LSPR of the aggregates and the absorbance at the wavelength of the LSPR of non-aggregated Au nanoprobes was used to evaluate the extent of aggregation.

### 3.1. Au Nanoprobe Functionalization

#### 3.1.1. Synthesis and Characterization of the 35 nm AuNPs Stock

There are many well-established methods to synthesize spherical AuNPs with reliable and repeatable results, enabling good control of their shape and size. Most of them are based on the chemical reaction between chloroauric acid (HAuCl_4_) and sodium citrate, imparting a negatively charged surface to the synthesized Au nanospheres [21]. In this work, we chose a seed-mediated growth method that provides AuNPs with the desired diameter, with excellent size dispersion [21].

The 35 nm AuNPs used herein had localized surface plasmon resonance (LSPR) bands centered around 525 nm (Figure S1), as expected for spherical AuNPs of this diameter [19,21]. The lack of secondary bands indicated the absence of aggregates. The sizes of the three batches of AuNPs from the UV-Vis spectra, calculated using the formula of Haiss et al. [19], were around 35 nm on average. The hydrodynamic diameter by intensity, obtained through DLS analysis (Table 2), was overall slightly higher compared with the one obtained from UV-Vis with the method of Haiss et al., as would be expected due to the influence of the citrate as a capping agent. The associated polydispersity (*Đ*) was in the 0.17–0.21 range, indicating good monodispersity of the stock suspension. As expected, the zeta potentials were negative and lower than −30 mV, indicating good colloidal stability (Table 2).

#### 3.1.2. Successful Functionalization of AuNPs

**Figure 2:**
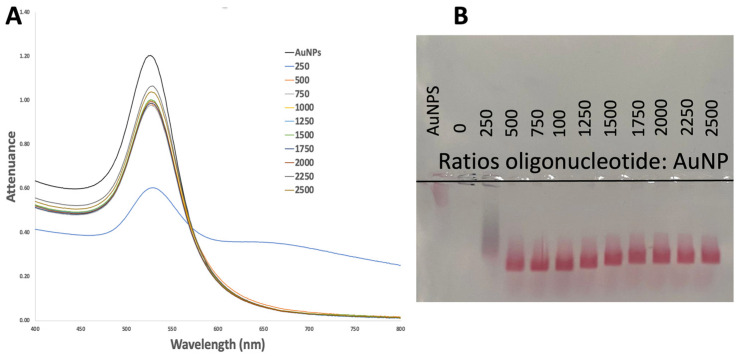
UV-Vis spectra (**A**) and agarose gel electrophoresis (**B**) of 35 nm AuNPs functionalized with the thiol-modified oligonucleotide (SH-C6-CCTTAATTCTCTTCGT) using molar ratios of oligo/AuNPs in the 250–2500 range in the presence of citrate/citric acid buffer with pH 3. All samples were dispersed in 10 mmol dm^−3^ phosphate buffer pH 8, except for the stock AuNPs, which are dispersed in a 2.2 mmol dm^−3^ trisodium citrate solution.

AuNPs of 40 nm were functionalized based on a pH method with different molar ratios of a thiol-modified 16-mer oligo nucleotide, and the state of aggregation of the resulting probes was assessed by UV-Vis spectroscopy (Figure 2A).

The spectra of the functionalized AuNPs are depicted in Figure 2. It is evident that at an oligonucleotide:AuNP ratio of 250 (Figure 2A), there was a noticeable decrease in extinction and/or the presence of a secondary extinction band at higher wavelengths, indicating an inefficient functionalization process. This inefficiency led to significant aggregation and/or loss of AuNPs. Conversely, for ratios equal to or greater than 500 (Figure 2A), the slight shift in the maximum extinction wavelength from 525 nm (citrate-AuNPs) to 527 nm (Au nanoprobe) can be attributed to the adsorption of oligonucleotides [5]. Furthermore, the shape and wavelength of the localized surface plasmon resonance (LSPR) band also suggest successful functionalization, as there was no evidence of aggregation or notable loss of AuNPs throughout the process. Hence, functionalization with oligonucleotide:AuNP ratios of 500 or greater appears promising, with no indications of aggregation observed in the resulting Au nanoprobes. These results are supported by agarose gel electrophoresis, where migration without signs of aggregation can be observed starting from ratio 500 (Figure 2B).

**Figure 3:**
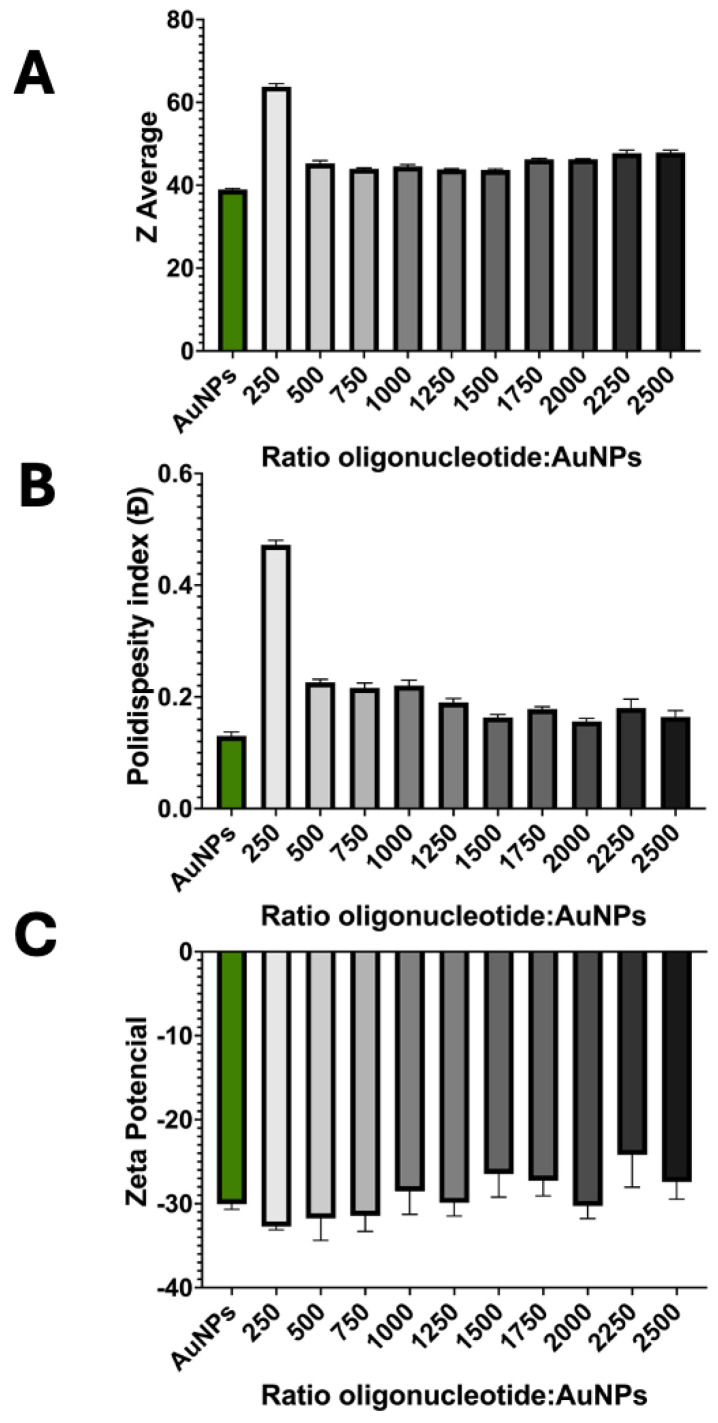
Physical–chemical characterization of AuNPs and the gold nanoprobes with oligonucleotides: AuNP ratios between 250 and 2500: zeta average (**A**) and polydispersity index (**B**) from DLS analysis and zeta potential (**C**) from ELS analysis. Data are presented as mean ± SD of at least three independent experiments.

DLS measurements confirmed the results from UV-Vis (Figure 3). The occurrence of aggregation can be seen at a oligonucleotide:AuNP ratio of 250, with a high polydispersity index (0.47) and a hydrodynamic diameter much higher than for the non-aggregated samples (63.41 nm). For oligonucleotide:AuNP ratios equal to or higher than 500, the hydrodynamic diameter presented values around 44–46 nm, all higher than the AuNPs stock, suggesting successful functionalization. The polydispersity index decreased with increasing ratios, reaching the desired polydispersity values below 0.2 at an oligos:AuNPs ratio of 1000, indicating a good monodispersity of the resulting probes prepared starting with this ratio or higher (Figure 3).

Considering these results, we selected an oligonucleotide:AuNPs ratio of 1000 for further studies. Ratios between 500 and 750 demonstrated low reproducibility in the functionalization and inconsistent results in the detection assay.

Thiol-modified oligonucleotides react with AuNPs through thiol/gold chemistry, but negative charges on both entities impair effective conjugation. Overcoming this requires challenging methods, particularly for larger AuNPs (>15 nm) [5,23,24,25,26]. The pH method used in our study proved to be optimal for the functionalization of larger, spherical AuNPs [5,22,23], as at a low pH, two of the nucleobases (A and C) are protonated, making DNA less negatively charged, thus reducing DNA-AuNP and DNA−DNA repulsions [23]. Compared to other methods like salt-aging, pH-assisted functionalization offers two key benefits: higher efficiency and shorter processing time, taking hours instead of days [22,23].

The oligonucleotide:AuNPs ratios selected in the current study were in the range of published data for the functionalization of spherical AuNPs. The ratio used for Au nanoprobes was expected to be higher compared with smaller 15 nm AuNPs, and was associated with a decrease in curvature, affecting the interactions among the DNA strands, and consequently affecting the DNA density/loading on the surfaces of the AuNPs compared to 15 nm AuNPs [5,24]. The ratio used in our current study was slightly smaller than the one used in our previous study, where a oligonucelotide:AuNP ratio of 1300 was used for the functionalization of 35 nm spherical AuNPs using pH-assisted functionalization [5]. However, since this method depends on protonation of specific DNA bases (A and C), variations in adenine and cytosine percentages between oligonucleotides may require different ratios. Additionally, their lengths are crucial for discrimination and stable Au nanoprobes, as shown previously [5,22].

### 3.2. Detection Assay

UV-Vis spectra of 35 nm Au nanoprobes were obtained in the presence (complementary/mutated/negative control) or absence (Au nanoprobe, blank) of DNA targets after adding MgCl_2_. When salt-induced aggregation occurred, as in the case of non-complementary DNA or the lack of any DNA target, a second plasmon band at higher wavelengths emerged. This occurred when non-complementary DNA targets did not hybridize with any part of the Au nanoprobe’s oligonucleotide, or when no DNA target was present, preventing protection against salt-induced aggregation. The second plasmon band at higher wavelengths was associated with a color change in the initial solution from red to blue-purple.

Aggregation of Au nanoprobes in the presence of DNA target occurs as the increase in the ionic strength induced by salt addition neutralizes the negative charges of the DNA phosphate on the surface of the AuNPs, thereby reducing the electrostatic forces between particles, reducing the interparticle distance, and promoting interparticle plasmon coupling with induced plasmon band shifts at higher wavelengths [27]. When there is complementarity between the oligonucleotides on the surfaces of the AuNPs and the DNA target, double-helix DNA structures are formed via hydrogen bonds between its bases that continue to be attached to the surfaces of AuNPs. These duplexes promote steric stabilization on the surfaces of AuNPs, and their repulsive forces contribute to the protection effect against salt-induced aggregation [28]. In this case, higher salt concentrations are necessary compared to non-complementary DNA targets to induce aggregation. Several types of salt can be used for the DNA detection methods based on aggregation of AuNPs. Yet, the use of multivalent metal ions is usually preferred as they are much more potent in charge screening and, therefore, aggregation of AuNPs by contributing more to positive charges compared to monovalent cations [27,29].

Complete hybridization of the DNA target with the Au nanoprobe’s oligonucleotide results in high resistance against aggregation, maintaining the probe’s initial red color and optical properties. A small shift in the plasmon band can occur due to the presence of hybridized DNA near the surface of Au nanoprobes.

UV-Vis spectra also provided information of the extent of aggregation, either directly by the extinction of the LSPR band of the aggregates or by calculating extinction ratios [5]. Ratios can be determined by comparing the LSPR maximum absorptions of non-aggregated and aggregated nanoparticles (AbsNon-Agg/AbsAgg). However, this approach disregards variations in aggregation patterns influenced by factors such as AuNP size, DNA target length, and the type of inducing salt. An alternative method involves subtracting the spectrum of the non-aggregated sample from that of the aggregated sample for each type of AuNP. This process identifies the minimum and maximum absorption wavelengths, which correspond to the locations of non-aggregated and aggregated peaks. Subsequently, these values are utilized to calculate AbsNon-Agg/AbsAgg ratios. Based on the UV-Vis spectra obtained in this study, differences in the localized surface plasmon resonance were found between Au nanoprobes incubated with normal complementary DNA and those incubated with mutated/deleted DNA for all tested conditions. The calculation of the AbsNon-Agg/AbsAgg ratio for each sample based on the UV-Vis spectra allowed us to mathematically evaluate the optimal conditions and to establish a limit of detection based on the statistical analysis of these ratios. The use of UV-Vis spectroscopy and the calculation of aggregation ratios has previously been proven to be an efficient tool in discrimination among DNA for non-crosslinking methods [5,6,30], with different ratios corresponding to differences in the aggregation profile. The hybridization temperature was set based on the melting temperature of the oligonucleotide probe to ensure optimal conditions for hybridization, with a perfectly complementary sequence to the exon 19 deletion mutation type [5,31].

**Figure 4:**
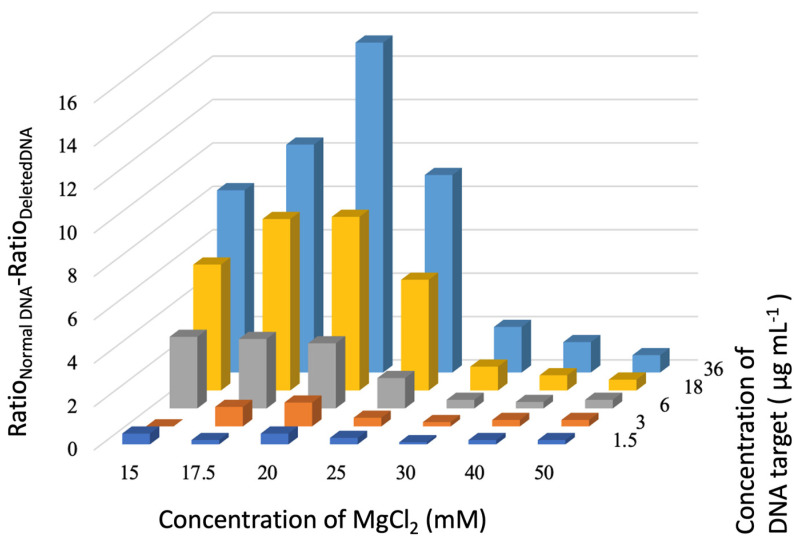
Ratio difference between AbsNon-Agg/AbsAgg ratio of normal DNA and deleted DNA at 1.5 (dark blue line), 3 (orange line), 6 (grey line), 18 (yellow line), and 36 (blue line) µg mL^−1^ and MgCl_2_: 15, 17.5, 20, 25, 30, 40, and 50 mM.

Figure 4 summarizes all results, with DNA targets at concentrations up to 36 µg mL^−1^ and a 7 MgCl_2_ concentration (from 15 to 50 mM). Each bar represents differences in AbsNon-Agg/AbsAgg ratios between assays with complementary normal DNA and non-complementary deleted targets, a measure of the discrimination of the assay. In Figure 4, we can observe a concentration-dependent discrimination between normal and mutated DNA, with an increase in the corresponding ratio difference occurring with the increase in the DNA target concentration, independent of the salt concentration used. This can be easily observed by the increasing size of bars from the front to the back of the graph. This would be expected, as the normal complementary DNA hybridizes with the Au nanoprobe, protecting against aggregation induced by MgCl_2_, while the mutated form does not hybridize. With the increase in the concentration of normal DNA targets, there will be more DNA strands available to bind to the oligonucleotide on the surfaces of the AuNPs due to base-pairing complementarity resulting in double-helix DNA structures that protect against salt-induced aggregation. In the case of mutated target DNA, even if there is an increase in the concentration and, therefore, there are more DNA strands available to bind to the oligonucleotide on the surfaces of the AuNPs, there is no base-pairing complementarity and, therefore, no hybridization on the surfaces of the nanoparticles. The effect is also dependent on salt concentration, as differences in aggregation ratios increase with increasing salt concentrations up to 20 mM. A higher salt concentration means higher cation concentrations available in the solution to neutralize the negative charge of the nanoprobes and induce aggregation [27]. For higher salt concentrations, there is a perceptible decrease in the difference in the ratios, as the duplexes formed between the Au nanoprobe and normal DNA target cannot overcome the increase in the ionic strength of the environment and its effect on the nanoparticles, therefore presenting a behavior closer to the Au nanoprobe incubated with a deleted DNA target. The effect of increasing salt concentration can be observed in the varying sizes of bars of the same color (same DNA target concentration) from the left to the right of the graph. An MgCl_2_ concentration of 20 mM is, thus, the optimal condition to discriminate between complementary and non-complementary DNA targets. This dependence of the discrimination between complementary and non-complementary DNA targets on the salt concentrations and on the DNA target concentration of the calculated AbsNon-Agg/AbsAgg ratios has already been acknowledged in previous studies [5,7,32]. This happens as both the hybridization and stability of the gold nanoparticles that are critical in these studies are influenced by several factors, including the target DNA, the type of salt used, and its concentration [27,32,33].

**Figure 5:**
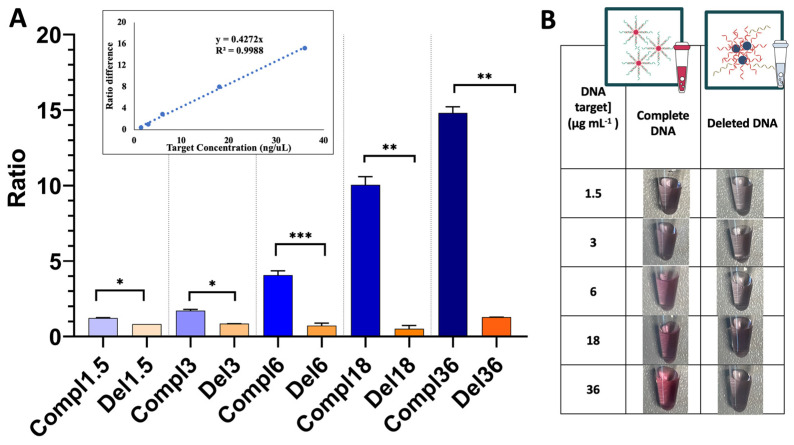
Analysis of Au nanoprobe incubated with target DNA at concentrations ranging from 1.5 to 36 µg mL^−1^ and 20 mM MgCl_2_. Representation of the AbsNon-Agg/AbsAgg ratio of normal/complementary DNA (blue bars) and deleted DNA (orange bar), one asterisk indicating *p* ≤ 0.05, two *p* ≤ 0.01, and three *p* ≤ 0.001 in cases of statistical significance (**A**). Dependence of the ratio difference (AbsNon-Agg/AbsAgg ratio of the Au nanoprobe incubated with normal DNA and the ratio of the same Au nanoprobe incubated with deleted DNA) on the target DNA concentration (inset). Photographs of the mutated Au nanoprobe and target DNA of different concentrations (1.5 to 36 µg mL^−1^) and 20 mM MgCl_2_ (**B**).

Figure 5 presents a complete analysis of assays performed with Au nanoprobes incubated with the target DNA at concentrations ranging from 1.5 to 36 µg mL^−1^, and at the optimal MgCl_2_ concentration of 20 mM. Statistical analysis (Figure 5A) using the unpaired *t*-test demonstrated that for all tested target DNA concentrations, the AbsNon-Agg/AbsAgg ratio of the Au nanoprobe in the presence of normal DNA was significantly different (*p* ≤ 0.05) compared with the AbsNon-Agg/AbsAgg ratio of the Au nanoprobe in the presence of deleted DNA (*p* ≤ 0.05). The unpaired *t*-test is a method used in inferential statistics to ascertain whether there exists a statistically notable distinction between the means of two independent groups [34]. Furthermore, discrimination is directly proportional to DNA target concentration (inset in Figure 5A), and is revealed by visual inspection of the color of the solution for DNA target concentrations higher than 6 µg mL^−1^ (Figure 5B).

Statistical significance for discrimination at all tested target DNA concentrations was observed for two other MgCl_2_ concentrations, one below (15 mM) and the other above (50 mM) the optimal 20 mM value (Figure S5). These results corroborate a higher protection against aggregation for Au nanoprobes incubated with complete/normal DNA compared with mutated/deleted DNA. For all conditions, the calculated ratios for the deleted DNA were very close to the values obtained for non-complementary DNA and the lack of DNA (Figure S4), indicating that they had similar aggregation profiles in the tested conditions. The low protection against aggregation, as in the case of deleted and non-complementary DNA noticed after addition of MgCl_2_, was associated with the lack of hybridization between the ssDNA on the surfaces of AuNPs and the DNA target presented in the solution. However, the behavior of normal/complete DNA differed across experimental conditions: The Au nanoprobe incubated with complete DNA consistently showed a higher ratio compared to mutated DNA in all tested conditions. Nonetheless, the ratio values varied significantly, suggesting different levels of protection against MgCl_2_-induced aggregation or inadequate aggregation. For example, when 15 mM MgCl_2_ was used, the difference between the ratios obtained for normal ssDNA and mutated ssDNA was low for all target DNA concentrations. Both normal ssDNA and mutated ssDNA showed high ratios, leading to poor discrimination between normal and mutated DNA sequences. The discrimination between the two types of DNA target became significant only at 6 ng/μL (Figure S5). This can be additionally confirmed by naked-eye observation: Even at the highest target DNA concentration tested (36 µg mL^−1^), the samples remained red. For MgCl_2_ concentrations higher than 20 mM, the ratio difference started to decrease. This decrease was due to aggregation in both normal and deleted DNA samples, leading to AbsNon-Agg/AbsAgg ratio values lower than 3. Likewise, it can be observed visually that the signal differentiation was also more difficult, becoming impossible for 50 mM MgCl_2_, where both samples were extensively aggregated already at the lowest target DNA concentration tested (6 µg mL^−1^) (Figure S5).

## 4. Conclusions

In this work, we describe the use of a 35 nm spherical Au–nanoprobe for the direct discrimination of a deletion mutation associated with non-small-cell lung cancer. Statistical analysis showed that when using a non-cross-linking method, this Au–nanoprobe is capable of successfully discriminating among complementary (normal) DNA and deleted or random non-complementary DNA targets, with a low discrimination limit of 1.5 µg mL^−1^. Statistical analysis of the AbsNon-Agg/AbsAgg ratio for Au nanoprobes incubated either with normal DNA or with deleted DNA showed there were significant differences above 1.5 µg mL^−1^ of target DNA when optimal conditions were used (20 mM MgCl_2_). This methodology has proven to be a reliable and robust screening technique that can be accurately used for DNA discrimination in controlled conditions. The results were obtained within a very short period (20 min), and the use of a spherical 35 nm Au–nanoprobe simplified integration in a disposable device for use at the point of care testing. Further optimization toward increased specificity in biological samples is still required before the technology can be translated to routine screening.

## Figures and Tables

**Table 1:** Sequences of the thiol-modified oligonucleotide used for AuNPs functionalization and of synthetic DNA targets.

Oligonucleotide	Length (bp)	Sequence 5′ to 3′
Thiol-modified oligonucleotide	16	**SH-C6-CCTTAATTCTCTTCGT**
Normal DNA	100	GAG TGT AGC TCC TAA AGG AAC AAC CGA AAA GCC TCT ACA **ACGAAGAGAATTAAGG** AAC TAT CGC TGC CCT TAA AAT TGA AAG AGT GGA AGA CCT AGG TCT
Deleted DNA	84	GAG TGT AGC TCC TAA AGG AAC AAC CGA AAA GCC TCT ACA **()** AAC TAT CGC TGC CCT TAA AAT TGA AAG AGT GGA AGA CCT AGG TCT
Non-complementary random DNA	84	CTTAGACCCTACAATGTACTAGTAGGCCTCTGCGCTGGCAATACAGATAAGATAATGTAGTCCCTGGCCTCAAAGGAACTCTCC

**Table 2:** AuNPs characterization through UV-Vis, DLS, and ELS analysis.

Batch	Diameter (nm)			Zeta Potential
	UV-Vis ^1^	DLS ^2^		
		Hydrodinamic Diameter ^2^	Polydispersity (Đ)	
1	37	38	0.19	−34.7 ± 1.3
2	36	41	0.21	−32.7 ± 1.0
3	35	36	0.17	−34.2 ± 1.5

^1^ Calculated using the formula of Haiss et al. [19]; ^2^ Hydrodynamic diameter presented as Z average by intensity.

## Data Availability

Data are presented in the article. Initial instrumental output data are available upon request from the corresponding author (M.E.).

## Supplementary Materials

The following supporting information can be downloaded at: https://www.mdpi.com/article/10.3390/bios14040162/s1, Figure S1: UV-Vis spectra of three different batches of the synthesized 35 nm AuNPs; Figure S2: UV-Vis spectra of three different batches of Au nanoprobes obtained with a oligonucleotide:AuNPs ratio of 1000; Figure S3: UV-Vis spectra analysis of the Au nanoprobe ratio 1000 incubated with MgCl_2_ at concentrations up to 25 mM; Figure S4: DNA concentration dependent effect of the AbsNon-Agg/AbsAgg ratio for 35 nm Au nanoprobes using three different targets: totally complementary (blue points and lines), deleted/noncomplementary (orange points and lines) and totally noncomplementary (purple points and lines) tested at different MgCl_2_ concentrations: 15 mM (A), 17.5 mM (B), 20 mM (C), 25 mM (D), 30 mM (E), 40 mM (F) and 50 mM (G); Figure S5: The bar graphs represent differences in AbsNon-Agg/AbsAgg ratios between complementary normal DNA (purple bar) and deleted/noncomplementary DNA (Orange lines) targets tested at different MgCl_2_ concentrations: at 15 mM (A) and 50 mM (B). One asterisk indicating *p* ≤ 0.05, two *p* ≤ 0.01, three *p* ≤ 0.001 and four asteriks indicating *p* ≤ 0.0001 in cases of statistical significance.
