# Supplementary material for: Gold Nanoprobes for Detection of a Crucial EGFR Deletion for Early Diagnosis of Non-Small-Cell Lung Cancer
Source: Biosensors (Basel). 2024 Mar 29;14(4):162. doi: 10.3390/bios14040162 (PMC11048279; doi:10.3390/bios14040162)
Supplement: Supplementary file 1 [file biosensors-14-00162-s001.zip › biosensors-2918266-supplementary.pdf]

biosensors

Article

Gold Nanoprobes for Detection of a Crucial EGFR Deletion for Early Diagnosis of Non-Small-Cell Lung Cancer

Maria Enea [1,*], Anupong Nuekaew [1], Ricardo Franco [2,3,*] and Eulália Pereira [1]

[1] LAQV /REQUIMTE, Departamento de Química e Bioquímica, Faculdade de Ciências, Universidade do Porto, Rua Campo Alegre, 687, Porto, 4169-007, Portugal; anue279@aucklanduni.ac.nz (A.N); eulalia.pereira@fc.up.pt (E.P)
[2] Associate Laboratory i4HB—Institute for Health and Bioeconomy, Faculdade de Ciências e Tecnologia, Universidade NOVA de Lisboa, 2819-516 Caparica, Portugal
[3] UCIBIO –Applied Molecular Biosciences Unit, Departamento de Química, Faculdade de Ciências e Tecnologia, Universidade NOVA de Lisboa, 2819-516 Caparica, Portugal
* Correspondence: menea@fc.up.pt (M.E.); ricardo.franco@fct.unl.pt (R.F.)

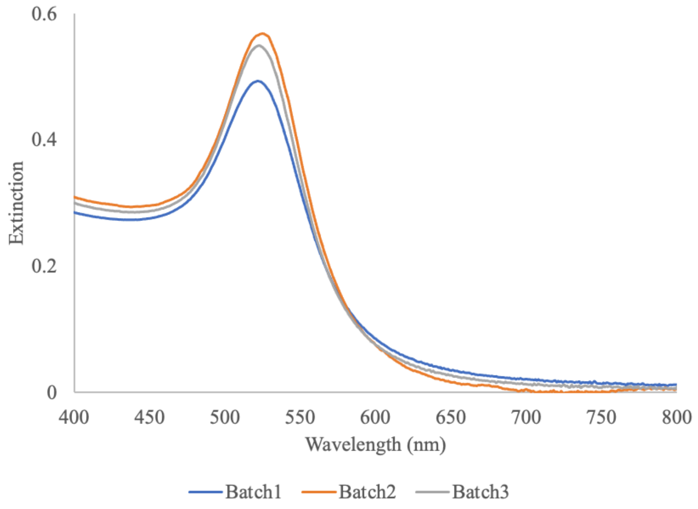

Figure S1. UV-Vis spectra of three different batches of the synthesized 35 nm AuNPs.

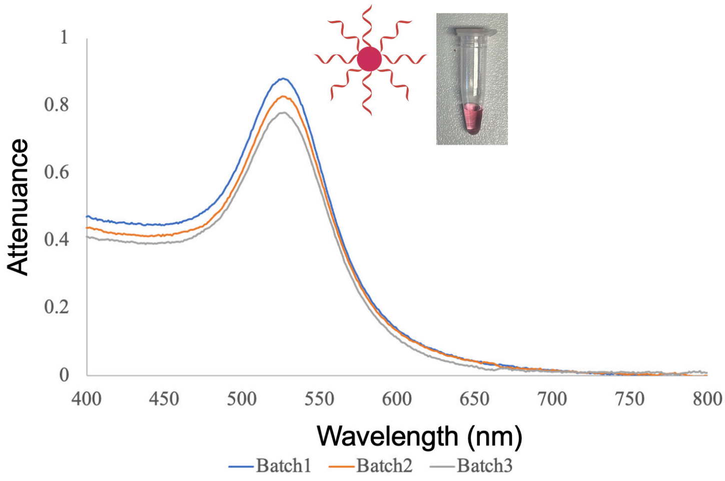

Figure S2. UV-Vis spectra of three different batches of Au nanoprobes obtained with a oligonucleotide:AuNPs ratio of 1000.

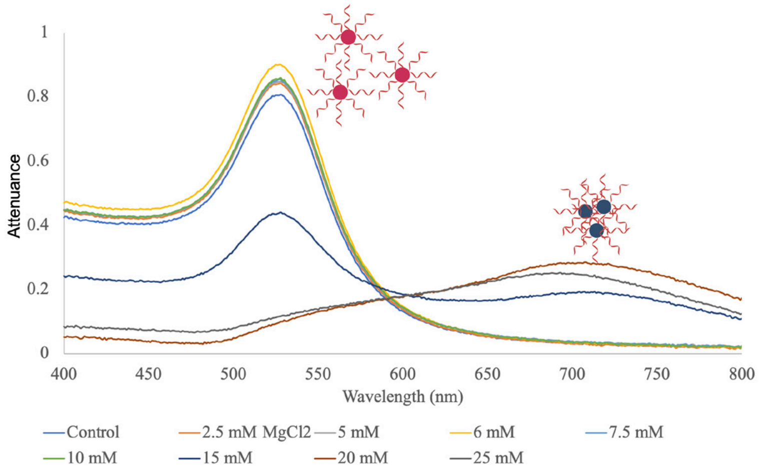

Figure S3. UV-Vis spectra analysis of the Au nanoprobe ratio 1000 incubated with $MgCl_2$ at concentrations up to 25 mM.

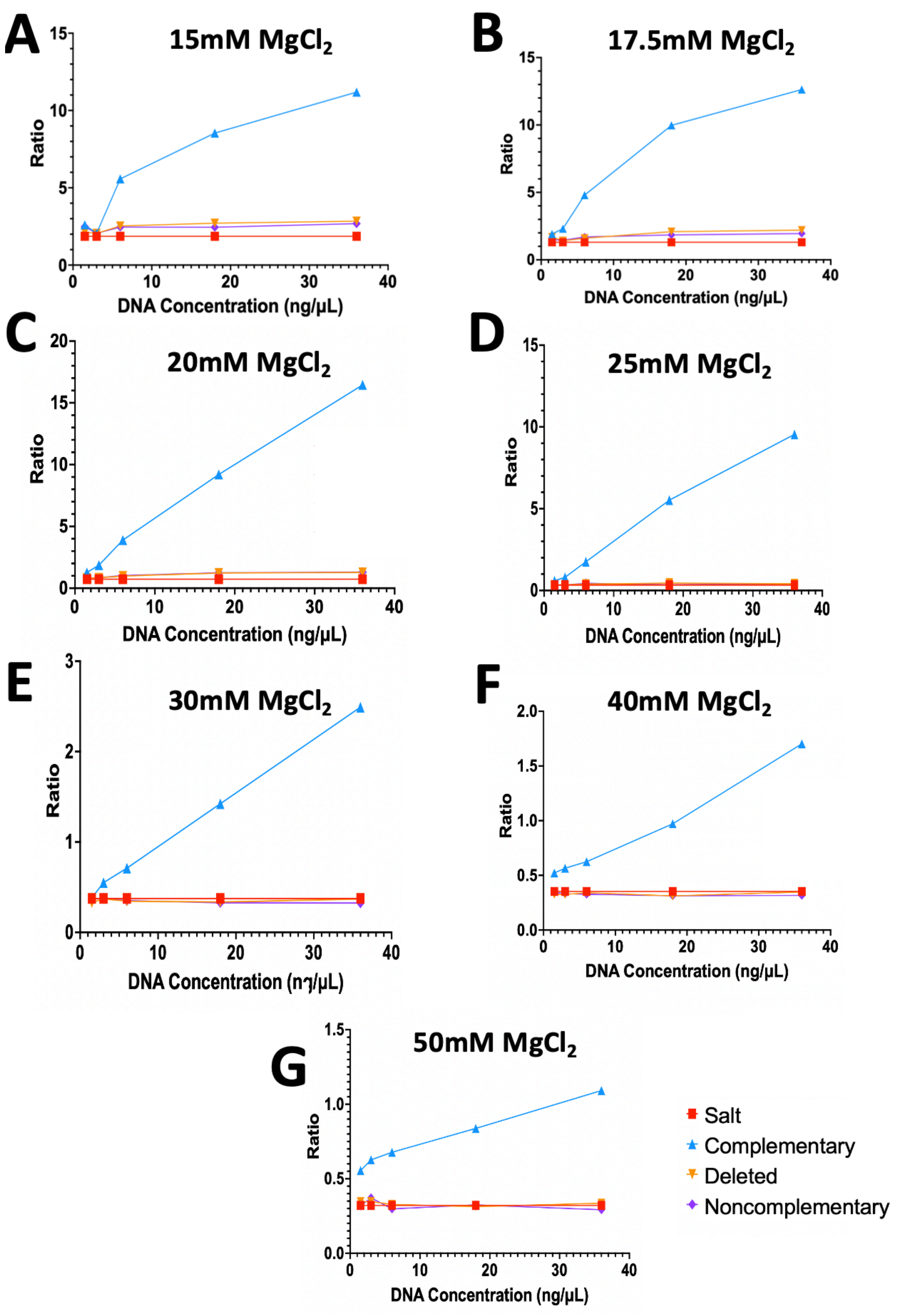

Figure S4. DNA concentration dependent effect of the AbsNon-Agg/AbsAgg ratio for 35 nm Au nanoprobes using three different targets: totally complementary (blue points and lines), deleted/noncomplementary (orange points and lines) and totally noncomplementary (purple points and lines) tested at different $MgCl_2$ concentrations: 15 mM (A), 17.5mM (B), 20mM(C), 25 mM (D), 30 mM (E), 40mM (F) and 50mM(G).

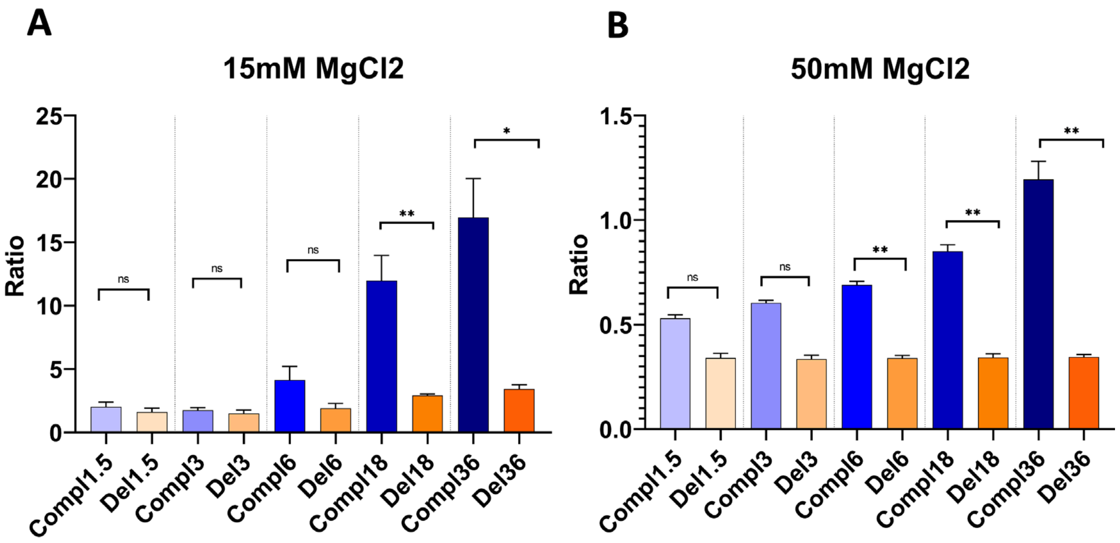

Figure S5. The bar graphs represent differences in AbsNon-Agg/AbsAgg ratios between complementary normal DNA (purple bar) and deleted/noncomplementary DNA (Orange lines) targets tested at different $MgCl_2$ concentrations: at 15 mM (A) and 50 mM(B). One asterisk indicating $p \leq 0.05$, two $p \leq 0.01$, three $p \leq 0.001$ and four asteriks indicating $p \leq 0.0001$ in cases of statistical significance.
